# Bullous Wells’ Syndrome: Case Report and Systematic Review

**DOI:** 10.3390/jcm14238370

**Published:** 2025-11-25

**Authors:** Giulia Ciccarese, Giorgia Sbarra, Giovanni Liguori, Gerardo Cazzato, William Andrew Rosato, Alexandre Raphael Meduri, Lucia Lospalluti, Aurora De Marco, Raffaele Filotico, Domenico Bonamonte, Francesco Drago, Caterina Foti

**Affiliations:** 1 Dermatology Unit, Department of Medical of Surgical Sciences, University of Foggia, Viale L. Pinto, 1, 71122 Foggia, Italy; 2 Section of Dermatology, Department of Precision and Regenerative Medicine and Jonian Area, University of Bari Aldo Moro, Piazza Giulio Cesare, 11, 70124 Bari, Italy; 3 Section of Molecular Pathology, Department of Precision and Regenerative Medicine and Ionian Area (DiMePRe-J), University of Bari Aldo Moro, Piazza Giulio Cesare, 11, 70124 Bari, Italy; 4 Casa di Cura Villa Montallegro, Via Monte Zovetto, 27, 16145 Genoa, Italy

**Keywords:** bullous Wells’ syndrome, itchy bullous lesions, eosinophilic cellulitis

## Abstract

**Background/Objectives**: Wells’ syndrome (WS) is an uncommon cutaneous disease with unknown etiology. Itchy bullous lesions and erythematous plaques characterize the bullous WS (BWS), a rare subtype of the syndrome. We describe the case of a woman affected by chronic lymphocytic leukemia who developed BWS and responded to the classic corticosteroid treatment. We also systematically reviewed the literature, analyzing the clinical, laboratory, and histological features and treatments of this rare disease. **Methods**: We used the databases Ovid MEDLINE, PubMed, and EMBASE with the following search terms: ((bullous Wells’ syndrome [MeSH Terms]) OR (eosinophilic cellulitis)) OR (bullous eosinophilic dermatitis) to identify and compare case reports of BWS. **Results:** We analyzed 28 patients, including our case. They were primarily female adults with a median age of 44.92 years. Blood eosinophilia was common, and histologically, the tissue samples showed an eosinophilic-neutrophilic dermal infiltrate. From a clinical perspective, the bullae were typically associated with or preceded by other lesions, primarily urticarial plaques, and mainly involved the extremities. Possible triggering agents of BWS include medications, insect bites, malignancies, and autoimmune/infectious diseases. Systemic steroids constituted the first-line treatment. Recent studies described the efficacy of the anti-interleukin-5 monoclonal antibody Mepolizumab in refractory cases of WS. **Conclusions:** Diagnosis of BWS is often challenging due to the rarity of the disease, clinical polymorphism, and multiple differential diagnoses. Integrating clinical features with laboratory and histopathological findings is essential for achieving a definitive diagnosis. Although the causal link between WS and underlying neoplastic/autoimmune/infective conditions is not always present, this possibility should be taken into account and investigated for the best patient management.

## 1. Introduction

Wells syndrome (WS) is a rare inflammatory cutaneous disease first described by George Wells in 1971 as a recurrent granulomatous dermatitis with eosinophilia. The disease is also known as eosinophilic cellulitis due to its clinical resemblance to bacterial cellulitis. It affects people of all races, primarily adults, without sex predilection [1,2].

The exact etiology is unknown: among the reported cases, some appear to be idiopathic, but others suggest the presence of triggering events, as long as underlying chronic disorders. Indeed, it is still debated whether WS is a distinct entity or a reaction pattern to different stimuli. The elements known to be triggering factors include: arthropod bites (mosquitoes, fleas, ticks and spiders), cutaneous and/or systemic infections (Parvovirus B19, Herpes simplex viruses, Mumps virus, Toxocara canis and others), drugs (antibiotics, nonsteroidal anti-inflammatory drugs, vaccinations, anti-tumor necrosis factor [TNF]-α and other biologic drugs), haematological and solid organ malignancies and autoimmune diseases (Churg-Strauss syndrome and ulcerative colitis). It has been hypothesized that these conditions can lead to an increased response and migration of eosinophils in the skin [1].

Clinical manifestations are heterogeneous, and histological appearance is typical but not specific. The limbs and the extremities are the site of predilection [1,2].

Seven clinical variants of WS have been categorized based on the prevalent elementary lesions observed during physical examination: plaque-type, annular granuloma-like, urticaria-like, papulovesicular, bullous, papulo-nodular, and fixed drug eruption-like. These variants of WS may present in succession or coexist in the same patient. Plaque-type unilesional presentation mimicking erysipelas has been reported to be more frequent in children than in adults. Itching and burning sensations typically precede the sudden onset of the skin lesions. Systemic symptoms, such as arthralgia and fever, may accompany the cutaneous eruption, which typically has a chronic, relapsing course. The prognosis is good because the disease may resolve spontaneously [1,2]. In adults, erythematous annular lesions resembling annular granuloma were the most frequently documented presentation of WS. The urticaria-like variant is also common and often concurrent with the plaque-type presentation. Other manifestations of WS, like bullae, papulovesicles, and papulo-nodules, are rarer [1,2].

The histological features of WS depend on the time of biopsy and the stage of the disease.

In the very early stages, tissue eosinophilia may be associated with sub-epidermal edema (acute stage, 2–3 days from onset). Later, the eosinophil degranulation is responsible for the development of the typical ‘flame figures’ corresponding to the collagen fiber degeneration caused by the release of toxic substances from the eosinophils (sub-acute stage, week 1–3). The subsequent migration of macrophages into the inflamed dermis and the appearance of giant cells around collagen deposits, produces cutaneous granulomatous infiltrates, eventually resulting in a gradual disappearance of the eosinophilia (regressive phase, 2–8 weeks) [3,4].

The clinical and histopathological heterogeneity of the disease constitutes a diagnostic challenge for both clinicians and pathologists.

The bullous variant is a very rare presentation of WS that is clinically characterized by itchy, tense vesico-bullous lesions often associated with erythematous plaques [4,5]. The disease is complex to diagnose and to distinguish from other bullous dermatoses, especially in the absence of specific laboratory and histological analyses.

In the present work, we describe the case of a woman affected by chronic lymphocytic leukemia (CLL) who developed BWS. In addition, to draw attention to this rare form of WS, we systematically reviewed the literature, analyzing its clinical, laboratory, and histological features, as well as the proposed treatments. To date, only single case reports and a few case series about BWS have been reported and a systematic review on this topic has never been published.

## 2. Materials and Methods

**Figure 1:**
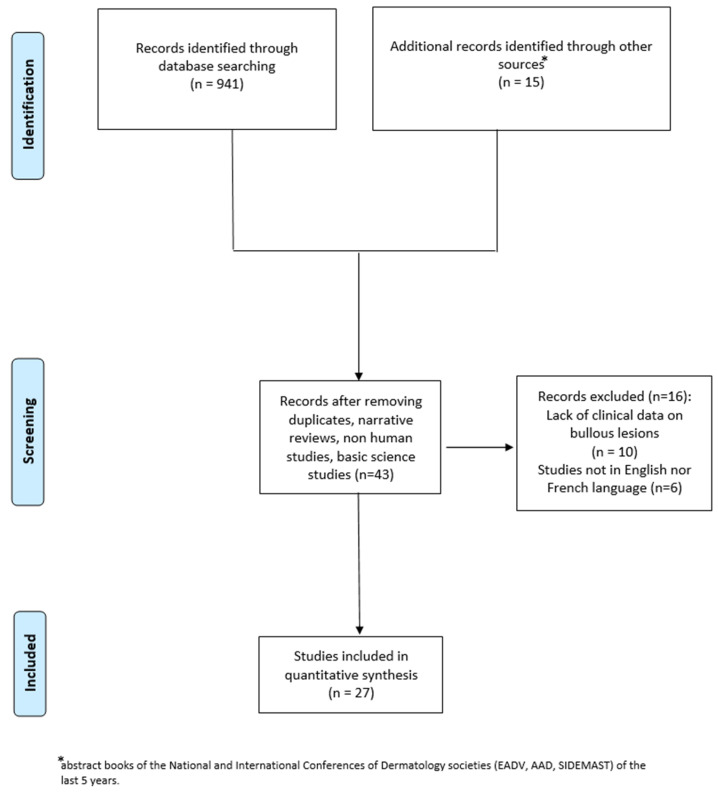
Selection of studies.

We performed a systematic electronic literature search following the PRISMA statement (www.prisma-statment.org, accessed on 27 October 2025), Table S1 [6]. Figure 1 presents a graphically altered PRISMA flowchart that includes all relevant information of study selection in a simple visual form. Articles were screened independently by two of us (W.A.R. and G.C) with any conflicts resolved by discussion, or involvement of a third assessor (C.F.) in the case of disagreement.

The last search was conducted on 17 August 2025; results were limited to English and French. We used the databases Ovid MEDLINE, PubMed and EMBASE with the following search terms: ((bullous Wells’ syndrome [MeSH Terms]) OR (eosinophilic cellulitis)) OR (bullous eosinophilic dermatitis) to find and compare reports of cases of BWS. Patients were defined as having BWS and included in our final review if all four of the following criteria were met: (I) signs and symptoms compatible with WS; (II) clinical description of bullous lesions of the skin; (III) laboratory and/or histologic data supporting the diagnosis; (IV) data on the used treatment/treatments. The review has been submitted to the PROSPERO dataset [ID 650076].

## 3. Results

### 3.1. Case Report

**Figure 2:**
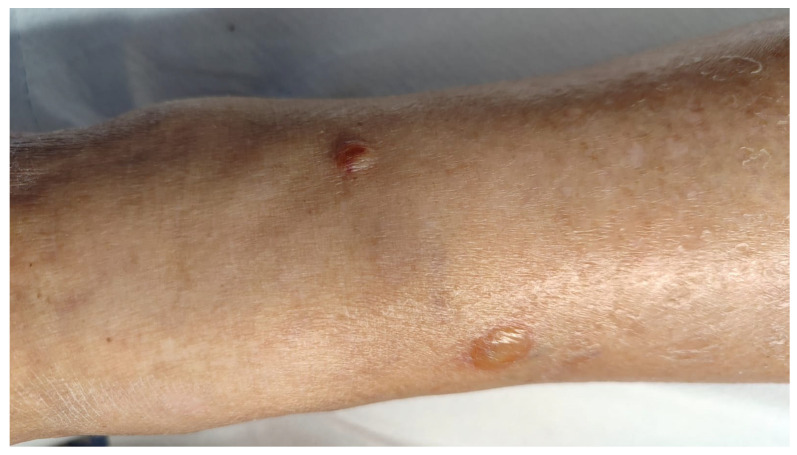
Bullous lesions of the right leg.

**Figure 3:**
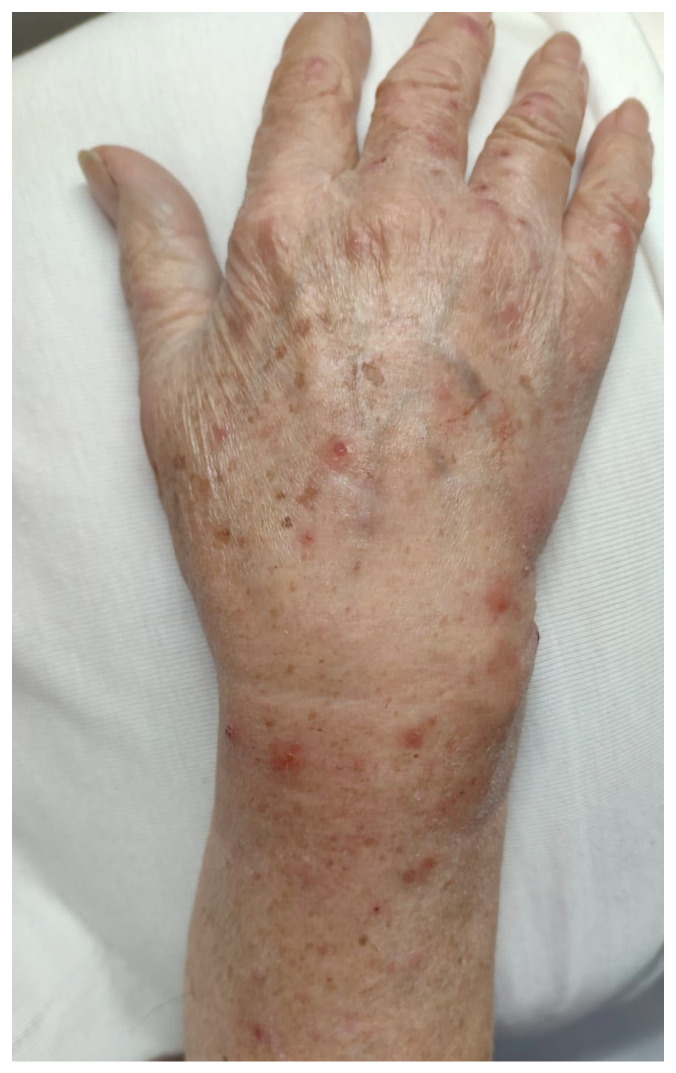
Erythematous papules and eroded vesicles on the dorsum of the left hand.

A 90-year-old female presented us with a one-year history of an itchy bullous skin eruption. The patient was affected by T cell CLL (STAGE 0) for which she was in haematological follow-up for the last 2 years. She had no personal family history of dermatological diseases and was otherwise in good health. She wasn’t undergoing any medical treatment. At the physical examination, vesicular, bullous lesions and crusts were observed on the upper and lower limbs. Isolated infiltrated erythematous plaques were also present on both hands and trunk. Laboratory investigations, including blood cell count, electrolytes, C-reactive protein, serum total IgE antibodies, antinuclear antibodies (ANA), anti-extractable nuclear antigen (ENA), anti-double-strand DNA, antibodies against bullous pemphigoid (BP)-180 antigen, and BP230 antigen, were within normal limits, as well as the anti-desmoglein-1 and 3 (Dsg1 and Dsg3) and anti-collagen VII antibodies. Two skin biopsies were performed, one from a bullous lesion of the right leg (Figure 2) and one from an eroded vesicle on the dorsum of the left hand (Figure 3).

**Figure 4:**
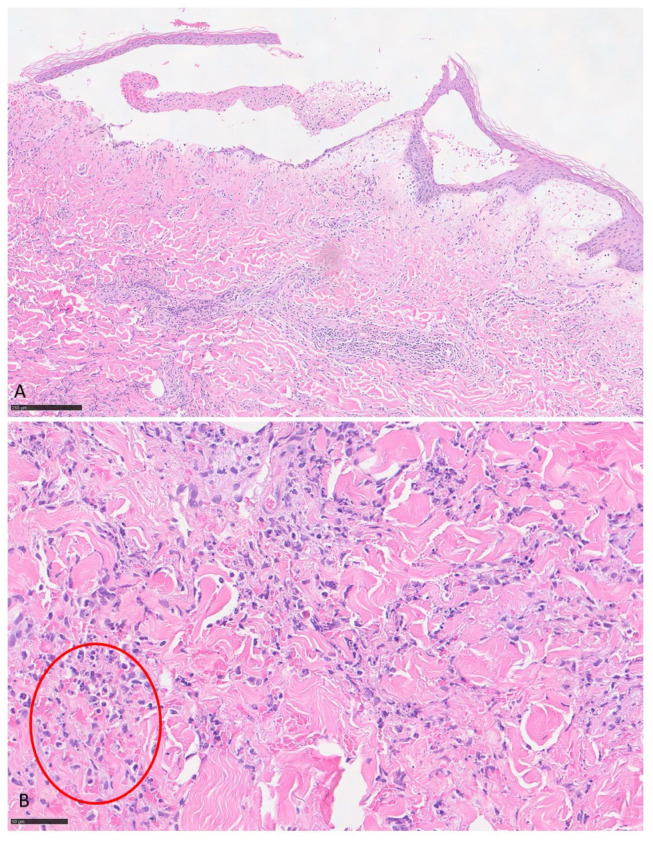
(**A**) Histology showed keratinocyte detachment in the spinous layer of the epidermis with formation of a fibrin-granulocytic eosinophilic blister (Hematoxylin-Eosin, 10× magnification); (**B**) a marked chronic lympho-eosinophilic inflammatory infiltration was observed in the perivascular area of the superficial and deep dermis (Hematoxylin-Eosin, 40× magnification); “flame figures” correspond to an amorphous eosinophilic structure composed of proteinaceous material and cellular debris, surrounded by degenerated eosinophils and histiocytes (red circle).

Histology of the first cutaneous sample showed keratinocyte detachment in the spinous layer of the epidermis with a fibrin-granulocytic eosinophilic blister; a marked lympho-eosinophilic inflammatory infiltration was observed in the perivascular area of the superficial and deep dermis. Histology of the second skin sample revealed epidermal vesiculation, focal erosion and marked eosinophilic granulocytic inflammatory infiltration with numerous “flame figures” in the mid-dermis. The histological picture of both samples supports the clinical diagnosis of WS” (Figure 4). Direct immunofluorescence was also performed providing negative results.

Based on the clinical features, laboratory investigations, and histological examination, BWS was diagnosed. After consultation with the hematologist, the patient received treatment with systemic corticosteroids (oral prednisone at an initial dose of 0.5 mg/kg/day), which showed benefits. At the three-month follow-up visit, the patient still presented a few eroded vesicles on the right hand; therefore, a new short cycle of systemic and topical steroids (mometasone furoate 0.1%) was prescribed. To date, the patient is still in follow-up and has not presented new lesions.

The patient gave written informed consent to publish her clinical, histological and laboratory data.

### 3.2. Systematic Literature Review

After reviewing the titles and abstracts of 956 articles from our initial search, we excluded 913 papers (duplicates, narrative reviews, non-human studies, basic science studies). After reviewing the full text of the remaining 43 articles, ten were excluded because they lacked detailed clinical data on bullous lesions, and six were not in English or French. We therefore analyzed 28 patients, including the case we described herein [3,5,7,8,9,10,11,12,13,14,15,16,17,18,19,20,21,22,23,24,25,26,27,28]. Clinical, laboratory, and histological features and treatments of the collected cases are summarized in Table 1.

The cases of BWS analyzed in our review have been published between 1988 and 2025, mainly by European authors. The majority of patients were female (71%) and the age of onset varied from one to ninety years, with a mean age of 44.9 years at diagnosis (range: 1–90 years). Only 5 cases (18%) referred to children under the age of twelve.

Blood eosinophil count, documented in every published case of BWS, was commonly increased; however, it was within normal ranges (0–530 cells/μL) in 36% of patients.

Conversely, the presence of the typical “flame figures” in the histological reports was almost always detected (75%).

The body areas most commonly affected by the bullous lesions were the upper and/or lower limbs: in 14 out of 28 cases (50%) only the limbs were involved, sometimes with unilateral presentation. A widespread location of the skin lesions was possible (43%), whereas the exclusive involvement of the face and/or neck was less frequent (7%).

The presence of mucosal manifestations was reported in only one of the 28 BWS cases, involving the tongue and throat.

A wide range of cutaneous lesions resulted associated with the bullae: plaques and nodules (75%), vesicles, papules, maculo-papules and purpuric lesions (Table 1). Itching was frequently reported (54%), whereas systemic signs and symptoms (fever or other flu-like symptoms) affected only one-fifth of patients (21%).

Comorbidities characterized by an infectious, inflammatory, or neoplastic etiology were documented in ten out of 28 patients (36%). In some cases of active diseases, they were considered as possible triggers for BWS (53%). More specifically, triggers were represented by infections (tinea pedis and onychomycosis, intestinal disease, upper respiratory tract infection), malignancies (CLL, colon cancer, small cell non Hodgkin’s B lymphoma, nasopharynx carcinoma), drugs (ustekinumab, lincomycin, thiopental, acetylsalicylic acid, pholcodine, tenoxicam, diclofenac sodium, amoxicillin), insect bites and autoimmune disease (Churg Strauss syndrome).

The time elapsed between the skin eruption and the potential trigger factor was highly variable, ranging from a few days for the insect bites to two years for the CLL.

The first-line treatment for BWS was represented by systemic steroids, as for the other variant of WS, in 86% of the patients, in some cases associated with the therapies administered for the underlying conditions (such as terbinafine for the *tinea pedis*) or with antihistamines and topical steroids in cases of diffuse skin eruption.

The disease often went into remission after treatment; however, in three patients, including ours, BWS had a relapsing course and required further treatment (Table 1 [3,5,7,8,9,10,11,12,13,14,15,16,17,18,19,20,21,22,23,24,25,26,27,28]).

## 4. Discussion

The diagnosis of BWS is often challenging due to the rarity of the disease and its similarity to other bullous dermatoses with hereditary, autoimmune, infectious, or drug-related etiologies. Indeed, the differential diagnoses of BWS include a wide range of cutaneous disorders such as BP, linear IgA bullous dermatitis, epidermolysis bullosa acquisita, bullous erysipelas, atypical herpes zoster, bullous scabies, bullous drug reaction with eosinophilia and systemic symptoms (DRESS), bullous Sweet syndrome, skin reactions to insect bites, porphyria, pseudoporphyria and others [2,7,29,30,31,32].

Diagnostic criteria for WS were proposed by Heelan et al. in 2013 [33]. These include major criteria (at least two out of four) and minor criteria (at least one). Major criteria are described as follows: (1) any of the reported clinical variants: plaque-type, annular granuloma-like, urticaria-like, papulovesicular, bullous, papulo-nodular, fixed-drug eruption-like; (2) relapsing, remitting course; (3) no evidence of systemic disease; (4) histology showing eosinophilic infiltrates, no vasculitis. Minor criteria include: (1) “flame figures” visible at histology; (2) granulomatous changes evident at histology; (3) peripheral eosinophilia not persistent and not greater than 1500/μL; (4) triggering factors (for example, drug intake) [33,34].

To our knowledge, the present work is the first comprehensive, systematic review of BWS. Indeed, the cases described to date were single case reports and small case series.

Notably, nine out of the 28 BWS cases reported to date were observed in Italy (32%), indicating a high level of awareness and knowledge of this rare disease in our country.

Blood eosinophilia was present to a slightly greater extent in BWS (64%) compared to all the other WS variants (50%) [2,3]. This difference may be explained with the acute nature of bullous lesions compared to other cutaneous manifestations (papules, plaques and nodules); therefore, the BWS cases might have been more frequently diagnosed during the acute or subacute phase, when the eosinophil level was usually high. However, our patient did not exhibit blood eosinophilia, likely because her bullous eruption was long-lasting and her disease in a subacute phase.

In our review, indeed, “flame figures” at histological examination occurred more frequently among BWS (75%) compared to other variants (50%), mainly indicating a subacute disease. As a matter of fact, in later stages the migration of macrophages into the dermis leads to the gradual disappearance of the tissue eosinophilia, to the absence of “flames figures” and to the formation of dermal granulomatous infiltrates [4,5,27]. Therefore, we can assume that the more frequent detection of “flame figures” in BWS compared to other WS forms may be related to the earlier execution of skin biopsy in the bullous variant of the disease.

As for clinical presentation, most BWS cases (75%) were associated with erythematous and sometimes urticarial, plaques and vesicles; skin lesions predominantly involved the lower and/or upper extremities (93%) and were often itchy (68%). The possible involvement of the oropharyngeal mucosa in patients with WS has been investigated only by a few authors, likely because few dermatologists are aware of its occurrence, as it may happen in the context of other dermatoses [30,35]. Notably, only one patient with nodular mucosal lesions of the tongue and throat, associated with diffuse skin lesions, has been reported [24].

In our review, possible triggering agents have been identified in about half of the patients, with variable timings from the trigger to BWS onset, ranging from a few days to 10 months. This aspect suggests that the association may sometimes be coincidental, although a causal link between the triggering factor and the development of BWS can be assumed when drug discontinuation led to resolution without subsequent relapses [9,22].

The association between BWS and malignancies has been documented in a consistent percentage of patients (14%). In some cases, the diagnosis of cancer and BWS were concomitant; in others, the malignancies preceded the eosinophilic disease by 1–2 years (Table 1). The pathogenic link between WS and hematologic diseases is not entirely understood. Some authors have speculated that in patients with an immunodeficiency due to hematologic disease, a trigger, such as a drug or a virus, induces cytokine production with an excess of interleukin (IL)-4 and IL-5, resulting in an altered immune response with eosinophil predominance [36,37]. One of the key events in the disease expression of WS is indeed the aberrant accumulation of eosinophils in the skin. Increased IL-5 levels observed in WS not only mobilize eosinophils from the bone marrow but also promote the homing of eosinophils by altering the expression of adhesion molecules. Tissue eosinophilia is commonly seen in cutaneous T-cell lymphoma. It has been hypothesized that the production of an eosinophil chemotactic factor from neoplastic lymphocytes may cause WS in patients with CLL [38]. Given the potential association with cancers, following a diagnosis of WS (and also BWS), we recommend obtaining an accurate medical history and conducting a detailed clinical examination to screen for underlying disorders through routine blood investigations. To date, an evidence-based malignancy screening protocol for WS patients that optimizes the risk-to-benefit ratio does not exist. Therefore, we propose that screening tests should be performed at the onset of eosinophilic disease, according to clinical findings, the patient’s medical history, age, and personal and family history of cancer [39].

Concerning the treatment, although WS may be limited to a restricted body area and may spontaneously remit, its widespread distribution and chronic-remitting course often require therapy. In all the cases of BWS that we reviewed, including our patient, systemic steroids constituted the first-line treatment, mainly administered orally but also intravenously and topically [5,7,8,10,11,12,13,14,15,16,17,18,19,20,21,22,23,24,25,26,27,28], as reported in the other variants of WS (Table 1). In patients suffering from recurrent disease, several steroid-sparing agents have been used, such as oral dapsone and cyclophosphamide.

Interestingly, only few cases of recalcitrant WS treated with Omalizumab (an anti-IgE monoclonal antibody) have been reported so far [40,41,42]. Recently, our research group documented the first patient with BWS who was successfully treated with this drug, achieving long-term remission [27]. Binding free IgE, Omalizumab downregulates the expression of the high-affinity IgE receptor (FcεRI) on mast cells and basophils. This receptor is expressed and upregulated also on skin-infiltrating eosinophils in eosinophilic conditions, such as atopic dermatitis and BP [43,44,45], which explains the successful treatment with Omalizumab in these diseases. Similarly, we can hypothesize that the overexpression of dermal eosinophils in WS upregulates FcεRI, which Omalizumab subsequently downregulates, leading to the clinical remission of the disease [41,42]. Moreover, the reduction in the level of different cytokines causing the recruitment, activation and survival of eosinophils (IL-5, IL-13, IL-4, IL-2, GM-CSF, IL-8) may also explain the anti-inflammatory efficacy of Omalizumab [44,45].

Recent studies have described the efficacy of another humanized monoclonal antibody in refractory cases of WS, specifically the anti-IL-5 Mepolizumab [46,47]. Indeed, this drug modulates IL-5, one of the central cytokines involved in eosinophil chemotaxis, maturation, and survival. Mepolizumab, therefore, acts to reduce eosinophil levels in the blood and lesional skin, thereby mitigating the cutaneous signs and symptoms of WS [46,47]. No cases of the bullous subtype of WS treated with Mepolizumab have been described to date; however, this treatment, together with Omalizumab, may represent a new therapeutic strategy in cases of relapsing disease, especially when it is necessary to limit the use of corticosteroids.

Mild and transient side effects have been occasionally reported following Omalizumab (slight fatigue and loose bowel movements a few days after injection [42]) and Mepolizumab treatment (headache, back pain, and reactions at the site of injection [47]). However, these adverse effects did not cause drug discontinuation. We therefore suggest that these monoclonal antibodies should be considered as second-line treatments in cases of WS that are refractory to first-line corticosteroid therapy or in cases where the side effects of corticosteroids outweigh their benefits.

## 5. Conclusions

Our article adds a comprehensive review of the clinical, laboratory, histologic, and therapeutic features of BWS to the current literature. Indeed, beyond single case reports and small case series, no systematic review has been published to date specifically addressing this topic.

In the presence of cutaneous bullous lesions, primarily when associated with other manifestations like erythematous papules, plaques and vesicles, after excluding common cutaneous bullous diseases (bullous erysipelas and BP), the diagnosis of BWS should not be neglected. Our review found that blood eosinophilia and the presence of “flame figures” in skin samples are more common in BWS compared to the other WS variants. Therefore, measuring total IgE in the patient’s serum and searching for the dermal eosinophilic infiltrate and “flame figures” in the skin tissue may help clinicians and pathologists achieve a definite diagnosis, which always derives from integrating the clinical features with laboratory and histopathological findings.

Although the causal link between WS and underlying neoplastic/autoimmune/infective conditions is not always evident, this possibility should be taken into account and investigated for the best patient management.

Our work may support the efficacy of systemic steroids as first-line treatment for BWS, but it also highlights that monoclonal antibodies targeting IgE and IL-5, key molecules involved in the pathogenesis of WS, are emerging as effective therapies for relapsing cases. Based on the results presented in our review, especially those regarding the most recently published case reports [33,46,47], we propose that Omalizumab and Mepolizumab may be promising treatment options for refractory WS, including its bullous subtype. Further studies on treating this rare disease with such drugs are warranted.

The limitations of case report-based systematic reviews include the fact that milder cases are less likely to be reported in the literature. Therefore, the sample size, especially in rare diseases like BWS, may be small, which limits the possibility of generalizing the study’s validity.

## Figures and Tables

**Table 1:** Clinical, laboratory, and histological features and treatments of the cases of BWS. WNR: within normal ranges; INR: information not reported; normal range for eosinophilic blood count: 0–530 cells/µL; 0–4.4%.

References	Country	Age	Sex	Blood Eosinophils (Cells/µL) (%)	Other Cutaneous Signs	Involved Sites	Skin Symptoms	Mucosal Involvement	Systemic Involvement	Comorbidities	Histological Flames Figures	Possible Trigger	Time from Trigger to Eruption	Treatment	Outcome	Follow up (Months)
Our patient	Italy	90	F	WNR	vesicles	Limbs	itching	no	no	chronic lymphocytic leukemia	yes	chronic lymphocytic leukemia	2 years	oral steroid (prednisolone 25 mg/day), mometasone furoate cream (twice daily)	relapsing disease then remission	6
Bao et al. (2025) [26]	Canada	52	F	1100	macules, papules	feet	itching	INR	INR	epilepsy and osteoarthrosis	yes	tinea pedis, onychomycosis	-	oral terbinafine (250 mg/day) for 3 months; ciclopirox cream, bilastine (40 mg twice day), prednisone(30 mg/day) for five days	remission	18
Ciccarese et al. (2025) [27]	Italy	36	F	1770 (18%)	urticarial plaques, vesicles	trunk, lower limbs	itching	no	no	no	yes	-	-	Oral steroid (prednisone 0.5 mg/kg/day), Omalizumab 300 mg/4 weeks	remission	36
Peckruhn et al. (2019) [8]	Germany	5	F	8850	plaques, nodules	Feet	Pain	INR	INR	INR	yes	Insect bite	14–21 days	Oral steroid (prednisolone 100 mg/day), dapsone (50 mg/day), dimetindene twice daily	remission	<1
Kim et al. (2023) [9]	Sud Korea	64	F	WNR	patches	Left limbs	Itching/burning sensation	INR	INR	Psoriasis	yes	Ustekinumab	10 days	Drug discontinuation	remission	INR
Papaetis et al. (2021) [28]	Cyprus	30	F	WNR	papules, nodules, plaques, urticaria, vesicles	left lower limb	itching	INR	fever	C. difficile intestinal infection	yes	intestinal infection	-	mometasone furoate cream (twice day), levocetirizine (10 mg day) for 1 month	remission	24
Guglielmo et al. (2020) [10]	Italy	82	M	WNR	patches, plaques, pustules	Limbs	INR	INR	INR	no	yes	-	-	Oral methylprednisolone (0.5 mg/kg/day)	remission	INR
Guglielmo et al. (2020) [10]	Italy	70	F	WNR	plaques	Limbs	Pain	INR	INR	Diabetes	yes	Insect bite	7 days	Oral methylprednisolone (0.5 mg/kg/day)	remission	INR
Lieberman JA (2017) [11]	Tennessee	11	M	730	patches, plaques	Lower limb	INR	INR	INR	INR	INR	Insect bite	2 days	Oral steroids, topical mupirocin	remission	INR
Feliciani et al. (2006) [7]	Italy	88	F	WNR	plaques, vesicles	trunk, folds, limbs	Itching/burning sensation	INR	INR	Colon carcinoma	INR	colon carcinoma	10 months	Oral methylprednisolone (40 mg/day)	remission	36
Lim et al. (2017) [12]	Singapore	44	F	810	papules, plaques, vesicles	face, trunk, upper limbs	Itching	INR	no	no	INR	-	-	Oral prednisolone (20 mg/day)	remission	6
Katoulis et al. (2009) [13]	Greece	64	F	700	plaques, vesicles	neck	Itching	INR	INR	Uterine fibromyomas, osteoporosis	no	-	-	Topical steroid	remission	12
Shams et al. (2012) [14]	Louisiana	11	F	3129 (21%)	plaques, erosions, crusts	face, neck, upper limbs	Itching	INR	Fever	no	yes	Insect bite	1 month	Intravenous steroid	INR	INR
Verma et al. (2012) [15]	India	40	M	WNR	plaques, vesicles	trunk, upper limbs	Itching	no	no	no	yes	-	-	Oral antihistamine, topical tacrolimus	remission	6
Kamiyama et al. (2015) [16]	Japan	39	F	1260 (15%)	plaques, vesicles, pustules	trunk, upper and lower limbs	Itching, tenderness	INR	no	no	yes	-	-	Oral prednisolone (0.5 mg/kg/day), fexofenadine 60 mg twice daily, topical steroid	remission	6
Soua et al. (2014) [17]	Tunisia	61	F	WNR	vesicles	upper limbs	Itching	no	no	no	yes	-	-	Oral prednisolone (40 mg/day), antihistamine 10 mg/day	remission	INR
Spinelli et al. (2008) [5]	Italy	73	F	1740 (14.3%)	purpura	lower limbs	Itching	INR	Fever, lymph node swelling	sigma adenocarcinoma 2 years earlier	yes	Small-cell non-Hodgkin’s B lymphoma	concomitant diagnosis	Oral methylprednisolone (20 mg/day), antihistamine, topical fusidic acid cream	remission	INR
Schuttelaar et al. (2003) [18]	Netherlands	55	M	5780 (34%)	plaques, vesicles, purpura	limbs	Itching, pain	INR	Malaise, fever, arthralgia	Nasal polyposis, asthma, peripheral eosinophilia	yes	Churg-Strauss syndrome	-	Intravenous dexamethasone (200 mg for 3 days), Doxycycline (100 mg twice daily), cyclophosphamide (150 mg/day)	remission	INR
Gilliam et al. (2005) [19]	USA	1	F	14,400 (48%)	plaques	lower limbs	Itching	INR	no	no	yes	-	-	Oral steroid (2 mg/kg), topical steroid	remission	12
Moon et al. (2013) [20]	Sud Korea	9	M	4770 (24.5%)	vesicles	trunk, limbs	Itching	INR	no	no	yes	Upper respiratory infection	21 days	Oral prednisolone (40 mg/day), cetirizine, dapsone, topical steroid	remission	INR
Caputo et al. (2006) [3]	Italy	64	F	(32%)	no	trunk, lower extremities	INR	INR	INR	no	no	-	-	Oral betamethasone sodium phosphate (4 mg/day), oral amoxicillin	remission	120
Caputo et al. (2006) [3]	Italy	34	F	(19%)	plaques	face	INR	INR	INR	no	no	-	-	Oral betamethasone sodium phosphate (4 mg/day), ceftriaxone sodium (2 g/day)	relapsing disease	60
Ferrier et al. (1988) [21]	France	42	F	1460 (8%)	plaques	face, trunk, upper limbs	Itching	INR	no	INR	yes	lincomycin, thiopental, acetylsalicylic c acid, pholcodine	-	Oral betamethasone (8 mg/day), dapsone	relapsing disease then remission	48
Seçkin et al. (2001) [22]	Turkey	28	F	990 (16.5%)	papules, vesicles, crusts	Limbs	Itching	INR	INR	INR	yes	tenoxicam and diclofenac sodium and/or amoxicillin	-	Oral prednisolone(40 mg/day)	remission	INR
Arca et al. (2007) [23]	Turkey	20	M	WNR	vesicles, pustules, plaques	limbs	Itching	INR	no	no	yes	-	-	Oral prednisone (60 mg/kg), tetracycline 500 mg twice daily	remission	12
Ling et al. (2002) [24]	UK	45	F	6180 (46.5%)	plaques	face, trunk, upper limbs	INR	INR	no	no	no	-	-	Oral prednisolone (30 mg/day), cetirizine (10 mg twice day)	remission	12
Ling et al. (2002) [24]	UK	42	M	WNR	plaques	face, limbs	Pain	tongue, throat	Influenza-like illness	INR	yes	-	-	Oral prednisolone(40 mg/day)	lost to follow-up	lost to follow up
Li M et al. (2025) [25]	China	58	M	750	papules, plaques, vesicles	neck, trunk, upper limbs	INR	INR	fever, lymph node swelling	nasopharynx carcinoma	yes	nasopharynx carcinoma	concomitant diagnosis	Oral prednisone (15 mg/day)	remission	INR

## Data Availability

Data available on reasonable request.

## Supplementary Materials

The following supporting information can be downloaded at: https://www.mdpi.com/article/10.3390/jcm14238370/s1, Table S1: “PRISMA checklist”.

## Abbreviations

The following abbreviations are used in this manuscript:

WS	Wells’ syndrome
BWS	Bullous Wells’ syndrome
IL	Interleukin
DRESS	Drug reaction with eosinophilia and systemic symptoms
BP	Bullous pemphigoid
